# How microorganisms use hydrophobicity and what does this mean for human needs?

**DOI:** 10.3389/fcimb.2014.00112

**Published:** 2014-08-19

**Authors:** Anna Krasowska, Karel Sigler

**Affiliations:** ^1^Department of Biotransformation, Faculty of Biotechnology, University of WroclawWroclaw, Poland; ^2^Department of Cell Biology, Institute of Microbiology, Czech Academy of SciencesPrague, Czech Republic

**Keywords:** cell surface, hydrophobicity, adhesion, pathogens, bioremediation

## Abstract

Cell surface hydrophobicity (CSH) plays a crucial role in the attachment to, or detachment from the surfaces. The influence of CSH on adhesion of microorganisms to biotic and abiotic surfaces in medicine as well as in bioremediation and fermentation industry has both negative and positive aspects. Hydrophobic microorganisms cause the damage of surfaces by biofilm formation; on the other hand, they can readily accumulate on organic pollutants and decompose them. Hydrophilic microorganisms also play a considerable role in removing organic wastes from the environment because of their high resistance to hydrophobic chemicals. Despite the many studies on the environmental and metabolic factors affecting CSH, the knowledge of this subject is still scanty and is in most cases limited to observing the impact of hydrophobicity on adhesion, aggregation or flocculation. The future of research seems to lie in finding a way to managing the microbial adhesion process, perhaps by steering cell hydrophobicity.

## Introduction

In the environment, microorganisms rarely live as planktonic cells and prefer growing as aggregates (named sometimes microbial granules Liu et al., 2004) which are often adsorbed at the air/liquid, air/solid and liquid/liquid interfacial surfaces. In aquatic or soil systems, microorganisms participate in self-immobilization processes (Wu et al., 2012) an important parameter of which is cell surface hydrophobicity (CSH). High CSH enables microorganisms to attach to hydrocarbon droplets on the surface or cells and to move from water to organic, hydrocarbon phase, where biosurfactants and enzymes decompose wastes (Kaczorek et al., 2008).

The rapid change of environmental conditions forces an adaptive modification in the microorganism which enhances its ability to survive. One of the many mechanisms involved in this process is the release of outer membrane vesicles (MV) in Gram-negative bacteria causing a significant increase in cell surface hydrophobicity, and an enhanced tendency to form biofilms (Baumgarten et al., 2012a). *Pseudomonas putida* strain DOT-T1E released MV within 10 min in the presence of toxic concentrations of long-chain alcohols, under osmotic stress caused by NaCl, in the presence of EDTA, and after heat shock (Baumgarten et al., 2012b). The release of bioactive MVs from cell surface is conserved across microbial life in bacteria, archea and fungi (Deatherage and Cookson, 2012) and these vesicular structures are used during interaction with host cells and tissues (Silverman et al., 2010).

The hydrophobic properties of microbial surfaces are conducive to adhesion to abiotic and biotic surfaces and to penetration of host tissues (Goulter et al., 2009; Rodrigues and Elimelech, 2009; Heilmann, 2011). Adequate hydrophobic/hydrophilic properties of microorganisms can contribute to useful processes such as degradation of hydrocarbons or biodegradable polyesters and during milk fermentation (Obuekwe et al., 2009). The development of specific adaptive mechanisms to the toxicity and low bioavailability of these substrates allows many bacteria to modify their cell surfaces regarding its hydrophobicity to permit direct hydrophobic-hydrophobic interactions with the substrates (Heipieper et al., 2010). On the other hand, some Gram-positive bacteria such as *Bacillus licheniformis* reduce the cell surface hydrophobicity in the presence of organic solvents and exhibit little affinity toward toxic organic compounds (Torres et al., 2011). Other Gram-positive bacteria such as *Mycobacterium frederiksbergense* can grow in the presence of anthracene (Wick et al., 2002; Yamashita et al., 2007). *Mycobacterium* sp. and *Rhodococcus erythropolis* have hydrophobic envelopes and show co-aggregation; this contributes to their being solvent-tolerant (de Carvalho et al., 2004).

Although adhesion can increase degradation of hydrocarbons, biodegradation does not necessarily require cell adhesion to the hydrocarbon phase (Abbasnezhad et al., 2011).

Unfortunately, in many instances microbial adhesion, aggregation and biofilm formation cause serious damage and diseases (Knobben et al., 2007; Auger et al., 2009; Ferreira and Zumbuehl, 2009).

## Are hydrophobic microorganisms more pathogenic?

The attachment of microbial cells to surfaces depends on a number of factors including, e.g., Brownian movement, van der Waals attraction, gravitational forces and surface electrostatic charges. One of the important factors is the hydrophobicity of the cells (Van Loosdrecht et al., 1990). Depending on the type of surface, hydrophobicity of cells can increase the propensity of microorganisms to adhesion. The more hydrophobic cells adhere more strongly to hydrophobic surfaces, while hydrophilic cells strongly adhere to hydrophilic surfaces (Kochkodan et al., 2008; Giaouris et al., 2009). However, one should take into consideration also the heterogeneity of microbial population. In the culture with planktonic, freely living microorganisms it is possible to observe the presence of both hydrophilic and hydrophobic cells, hence only part of them participate in the adhesion. Another important tenet is that microorganisms can switch between hydrophobic and hydrophilic phenotypes in response to changes in environmental conditions (temperature, composition of nutrients, etc.) and growth phases (Borecká-Melkusová and Bujdaková, 2008; Bujdakova et al., 2013).

Considering that medical implants such as catheters, mechanical heart valves or pacemakers are constructed from hydrophobic materials (silicon, stainless steel, teflon, etc.), hydrophobic microorganisms are relatively easily adhering to them. One of the solutions of this problem is using implants from anti-biofilm materials that can delay or completely avoid the adhesion of microorganisms. For example, the use of polymeric nanofibers on polystyrene surface significantly delayed bacterial and fungal biofilm formation (Arciola et al., 2012; Kargar et al., 2012). Natural macromolecules (e.g., gluten, silk fibroin, and fibrinogen) can be more resistant to the bacterial or fungal colonization due to their lower hydrophobicity and can be used with success in tissue engineering (Ma, 2008). Another strategy of preventing surfaces from bacterial colonization is the modification of surfaces by coating them with noble metals, i.e., silver nanoparticles (Menno et al., 2011). The silver nanoparticles can be either deposited directly on the surface of medical devices, or applied in a polymeric surface coating. The exact mechanism of antimicrobial action of silver is still not completely known (Menno et al., 2011).

Biofilm formation on tissues is another medical problem because of the strong resistance of these microbial structures to drugs (Palmer et al., 2007; Murzyn et al., 2010; Archer et al., 2011). Adhesion is the first step to colonization of tissues and the prevention of this process seems to be a good strategy for keeping good health. CSH is an important feature of the adhesion (Hazen, 2004); hence considering the possibility of regulating this element of pathogenesis is worth the commitment.

Microorganisms in suspension may form co-aggregates which then adhere to surfaces (Bos et al., 1999). Gibbons and Nygaard (1970) observed that certain pairs of planktonic oral microorganisms underwent fast and extensive co-aggregation whereas others did not. Other investigators have demonstrated that each microbial strain or species has its own specific co-aggregation partner (Kolenbrander, 1989). This co-aggregation process is mediated by stereo-chemical interactions between specific surface components and the interacting microbial cell surfaces, such as lectin-carbohydrate interactions (Cisar et al., 1979; Kolenbrander, 1989; Kolenbrander and London, 1992).

Despite individual cell hydrophobicity determinants characteristic for each species, several compounds such as lipoteichoic acid, outer membrane proteins and lipids, surface fibrils, various fimbriae or core oligosaccharides are suggested to be common bacterial CSH features (Table 1).

**Table 1:** **Factors involved in CSH and adhesion of bacteria and fungi**.

**Factor**	**Bacteria/fungi**	**References**
S-layer	*Bacillus, Lactobacillus*	Sidhu and Olsen, 1997; Auger et al., 2009
Lipoteichoic acid	*Streptococcus, Staphylococcus*	Morath, 2005; Xia et al., 2010
Outer membrane protein	*Pseudomonas, Escherichia*	Akama et al., 2004; Tokuda and Matsuyama, 2004
Surface fibrils	*Streptococcus, Streptomyces, Candida*	Hazen and Hazen, 1993; McNab et al., 1999; Claessen et al., 2003
Oligosaccharides	Enterobacteriaceae: *Legionella, Escherichia, Salmonella*	Zähringer et al., 1995; Heinrichs et al., 1998; Frirdich and Whitfield, 2005
Hydrophobins	Filamentous fungi: *Aspergillus, Candida*	Linder, 2009
Volatile organic compounds	*Fusarium*	Vergara-Fernández et al., 2006; Minerdi et al., 2009
β-(1,3)-D-glucans	*Candida*	Fukazawa and Kagaya, 1997
Adhesins	*Candida, Escherichia, Streptococcus*	Brauner et al., 1990; Higashi et al., 1998; Dea et al., 2000; Rauceo et al., 2004

In enteric bacteria, adhesion to host cells is often promoted by a lectin found on surface-localized fimbriae (Isberg and Barnes, 2002) that have been found to contain a high proportion of hydrophobic amino acid residues (Rosenberg and Kjelleberg, 1986). Fimbriae play a role in cell surface hydrophobicity and attachment, probably by overcoming the initial electrostatic repulsion barrier that exists between the cell and the surface (Corpe, 1980). Gram-positive microorganisms and their strategies for establishing adhesive contact with the endothelium involve extracellular matrix proteins which act as colonization bridges with host cells (John et al., 1999). Recently, Gram-positive fimbriae have started to be uncovered (Ton-That and Schneewind, 2004) and, as in Gram-negative pathogens, Gram-positive fimbriae seem to play an important role in the adhesion of bacteria to host surfaces (Pizarro-Cerda and Cossart, 2006). Besides fimbriae, different bacterial nonpolymeric adhesins exist which recognize many different elements of host-cell surfaces (collagens, laminins, elastin, proteoglycans). Adhesive glycoproteins such as vitronectin, fibrinogen, and specially fibronectin, which can be secreted or associated with plasma membrane, are recognized by many different species of bacterial pathogens. The example is *Staphylococcus aureus* which expresses fibronectin-binding proteins that present mechano-functional properties (Schwarz-Linek et al., 2003).

In contrast to factors governing bacterial adhesion, the factors responsible for adhesion of cells of fungal mycelia are still poorly known. Some important factors connected with pathogenesis, adhesion of and colonization by indoor molds include β (1,3)-D-glucans, outer cell wall fungal hydrophobins and volatile organic compounds such as aldehydes, aromatic compounds and amines (McGinnis, 2004; Pieckova, 2012). The function of hydrophobins is the coordination of adherence between hyphae and macroorganism (Linder, 2009). Hydrophobicity is also due to other proteins functioning as repellents, which were detected in pathogenic fungi (Feofilova, 2010). More detailed information is available about hydrophobicity, adhesion and infections caused by the opportunistic yeast pathogen *Candida albicans* (Sanglard et al., 2009; Fortuna et al., 2012). Hydrophilic cells have an elongated acid-labile mannan fraction in the cell wall and the length of this structure affects the folding of cell wall fibrils and the cell surface hydrophobicity (Netea et al., 2008) (Table 1).

There are several proteins closely connected with CSH that affect adhesion of *C. albicans* to surfaces. The first described hydrophobic protein in the surface of *C. albicans* was CSH1p, and Csh gene deletion resulted in 75% reduction of surface hydrophobicity and reduction in adhesion to the extracellular matrix protein fibronectin (Singleton et al., 2005). Other results suggest that CSH1p phenotype has a pleiotropic nature and its contribution to pathogenesis (e.g., adhesion to fibronectin) is independent of reduced cell surface hydrophobicity (Singleton et al., 2005).

Several cell surface adhesins such as those belonging to the agglutinin-like sequence (ALS) family increase yeast aggregation and adhesion to epithelia (Dranginis et al., 2007; Aoki et al., 2012). Beaussart et al. (2012) postulated that the hydrophobicity of hyphae depends on enhanced exposure of Als3 protein on the surface and causes stronger *C. albicans* adhesion to hydrophobic substrata (Table 1).

If hydrophobic cells can more easily and faster capture new places for living and nutrient uptake, why do hydrophilic cells also coexist? This question is especially justified for hydrophilic microorganisms living on inner or outer human body surfaces, which have mostly hydrophobic character (Gilbert et al., 1991; Feingold, 2007; Linden et al., 2008).

It should be first noted that the attachment to surfaces depends not only on the hydrophobicity of cells; factors responsible for this process include also Brownian movement, Van der Waals attraction, gravitational forces and surface electrostatic charges (Van Loosdrecht et al., 1987).

In general, two physico-chemical approaches describe microbial adhesive interactions. The thermodynamic approach is based on the assumption that microbial surfaces physically contact each other under conditions of thermodynamic equilibrium (Absolom et al., 1983; Busscher et al., 1984). This thermodynamic approach takes into account the role of surface free energies but does not include the role of electrostatic interactions. An alternative approach is the classical DLVO (Derjaguin and Landau, Verwey, and Overbeek) theory which describes the interaction energies between the interacting surfaces and uses Lifshitz-van der Waals and electrostatic interactions and their decay with separation distance (Rutter and Vincent, 1980; Tadros, 1980).

One of explanation of the role of hydrophilic cells invokes as interaction between hydrophilic and hydrophobic surfaces in specific conditions. In pure water, repulsive forces are often observed between hydrophobic polystyrene and hydrophilic surfaces. By contrast, Thormann et al. (2008) found a typically DLVO-like interaction between hydrophobic polystyrene particles and a hydrophilic surface that was regulated by an addition of salt. In its presence, secondary adhesion processes were observed and the loosely bound polystyrene molecules were bridging to the surface (Cerca et al., 2005). Accordingly, when the concentration of NaCl increased, the Debye lengths decreased (Cerca et al., 2005).

Thus, though the primary adhesion of a hydrophobic particle to the hydrophilic surface is weak, the bridging polymers give rise to long-range attraction which will effectively anchor the particle to the surface.

Microbial adhesion to surfaces involves also physico-chemical phenomena, which can effectively mask the influence of CSH as a biological factor. The notions about the lack of direct relationship between CSH and the ability to adhere to either a hydrophilic or hydrophobic substrate were explored in, e.g., *Staphylococcus epidermidis* (Cerca et al., 2005). Another pathogenic bacterium, *Staphyloccocus aureus*, was found to attach to both hydrophobic indium tin oxide (ITO)-coated glass and hydrophilic glass surfaces, with stronger adhesion to hydrophobic surface (Zmantar et al., 2011). As documented by the adhesion of *Brukholderia* strains to *n*-hexadecane, the microorganisms can be able to adapt to the presence of hydrocarbon by modifying their cell surface composition to increase or decrease adhesion (Chakarborty et al., 2010).

Despite the development of various methods of recognition of hydrophobic properties of microorganisms, the above examples indicate that the knowledge about the processes of adhesion in microbiology is still insufficient. Cell surface properties have been difficult to study at the subcellular level, especially on hydrated, live cells such as tissues.

The ability to regulate the CSH allows microorganisms to either promote or hinder attachment (Rosenberg and Kjelleberg, 1986); hence hydrophilic microorganisms can be hypothetically considered as transitional forms on their move through water environment to places of colonization and pathogenesis.

## Should we value and use microbial hydrophobicity?

On the one hand, hydrophobic microorganisms are more invasive and cause diseases difficult to treat (Doyle, 2000). On the other hand, the positive role of hydrophobic cells can be utilized in cleaning up the environment from aromatic and xenobiotic organic compounds. Various environmental contaminants, such as toluene, are highly hydrophobic and toxic for cells by causing disruption of the plasma membrane. Hydrophobic microorganisms tend to accumulate on such pollution compounds and decompose them (Kobayashi et al., 1999). On the other hand, hydrophilic microbes are often more resistant to the toxic effects of solvents due to the modification of the lipopolysaccharides in the bacterial outer cell membrane, which protects them from the attachment of organic molecules (Kobayashi et al., 1999). Solvent-resistant microorganisms are also capable of mineralization of toluene (Heipieper et al., 2007). For these reasons, their use could be considered to prevail over the hydrophobic microorganisms. In a study of migration through soil, hydrophobic bacteria were found to be slower compared to hydrophilic bacteria (Stevik et al., 2004). In bioremediation processes, the fast dispersion of inoculated microorganisms is desirable and hydrophilic strains with low tendency to adhesion are therefore advantageous (Obuekwe et al., 2009).

Several bacteria with low cell surface hydrophobicity develop resistance to solvents by undergoing modification of the lipopolysaccharide or porines of the outer membrane. The solvent-tolerant microorganisms are the first to colonize and become active in the removal of pollutants (Megharaj et al., 2011). The application of solvent-tolerant bacteria or modification of these bacteria with an appropriate catabolic potential can provide advantages in bioremediation programs.

Biological wastewater treatment is among the most important biotechnological applications. CSH plays an indispensable role also in wastewater treatment in microbial granular reactors, where microorganisms aggregate in aerobic and anaerobic granules. The formation and structure of biogranules are associated very closely with cell hydrophobicity. With increasing cell hydrophobicity, cell-to-cell adhesion and aggregation is observed to grow (Liu et al., 2009) and degradation of wastes such as phenol, pyridine and its derivatives, nitrogen and phosphorus compounds or heavy metals via various metabolic pathways occurs (Adav et al., 2008). One of the problems is the slow formation of the granules, which takes many weeks. Some researchers indicated that application of cultures of microorganisms with high CSH accelerates the formation of the granules (Adav et al., 2005). Though the search for the universal strain(s) for microbial granulation is often directed at finding the most hydrophobic microorganisms present in the experimental biomass, experiments proved that an enrichment of microbial granules with selected, strongly hydrophobic strains was insufficient and other properties of microorganisms are also important in wastewater treatment (Ivanov et al., 2006). Amyloid proteins (fimbriae or other microbial surface-associated structures) are expressed by many types of bacteria in biofilms from various habitats (Larsen et al., 2008). Amyloid proteins play an important role in activated sludge wastewater treatment plants. A broad range of phylogenetically distant species in the phyla *Proteobacteria, Bacteroidetes, Chloroflexi, Firmicutes*, and *Actinobacteria* produce amyloids in the sluge (Larsen et al., 2007). The function of amyloid fibrils is assumed to be related to enhanced adhesion to surfaces and biofilm formation (Prigent-Combaret et al., 2000). Several filamentous bacteria in Danish wastewater treatment plants such as *Meganema perideroedes, Gordona amarae* and *Skermania piniformis* are known to be highly hydrophobic (Iwahori et al., 2001; Nielsen et al., 2001; Kragelund et al., 2006), which might be a consequence of the presence of amyloid-like material.

In the food industry, typical materials which are used in installations are stainless steel, rubber and polytetrafluoro-ethylene (PTFE). Sinde and Carballo (2000) demonstrated that hydrophobic pathogens such as *Salmonella* spp. and *Listeria monocytogenes* easily attach to these materials. Microbiological contamination costs the food industry many millions of dollars annually (Brooks and Flint, 2008) and the research into the processes of microbial adhesion is therefore extremely important. However, many researchers who conduct investigations on the relationships between hydrophobicity of microbial surfaces and adhesion to different materials conclude that it is very difficult to evaluate the results, since many parameters are involved in these processes in interfacial systems (Brooks and Flint, 2008). Like in the case of medical implants, surfaces of materials used in the food industry can be modified. For instance, ions lowering surface energy can be implanted to stainless steel, bioactive surfaces with immobilized enzymes can be generated or antimicrobial chemicals can be used in the coating of surfaces (Yazdankhah et al., 2006; Zhao and Liu, 2006; Srinivasan and Swain, 2007; Tabak et al., 2007).

Microbial hydrophobicity plays an important role in processes such as food production, spoilage, etc. due to interactions between microorganisms and food components such as lipids and proteins. For example, species of lactic acid bacteria such as *Lactococcus lactis* subsp. lactis biov. diacetylactis, which have a key role in the production of yogurts, cheese or sausages, could influence and change the stability of food emulsions. Bacteria with more hydrophobic surfaces have a higher affinity for milk fat and aroma compounds. Ly et al. (2008) observed that lactic acid bacteria led to the adsorption on lipid droplets but did not cause a destabilization of the emulsion, but when the bacterial surface charge was opposite to the one of the emulsion, the droplets the emulsions made with ionic surface-active compounds were unstable (Ly et al., 2006).

## Concluding remarks

Hydrophobicity of microorganims seems to have a considerable function in many fields of human activities and health. Hydrophobic cells play a key role in the formation of biofilms or removing contaminants from soil and water, but some results indicate that hydrophilic microorganisms also have an important role in these processes. Despite the many studies on the environmental and metabolic factors affecting CSH, the knowledge of this subject is still scanty and is in most cases limited to observing the impact of hydrophobicity on adhesion, aggregation or flocculation. The future of research lies in the determination of microbial strains and constructing the hydrophobic/hydrophilic profile of populations in bioremediated environments. On the other hand, in the prevention of microbial adhesion to implants or tissue one should pay attention to hydrophilic cells moving to the surfaces for their settlement, and control their quantity in the body using suitable drugs.

### Conflict of interest statement

The authors declare that the research was conducted in the absence of any commercial or financial relationships that could be construed as a potential conflict of interest.
